# Video-based analysis of the blink reflex in Parkinson’s disease patients

**DOI:** 10.1186/s12938-024-01236-w

**Published:** 2024-04-23

**Authors:** Talisa S. Jansen, Gökhan Güney, Bergita Ganse, Mariana H. G. Monje, Jörg B. Schulz, Manuel Dafotakis, Christoph Hoog Antink, Anne K. Braczynski

**Affiliations:** 1 Department of Neurology, RWTH University Hospital, Aachen, Germany; 2 KIS*MED (AI Systems in Medicine Lab) Technische Universität Darmstadt, Darmstadt, Germany; 3 Innovative Implant Development, Saarland University, Homburg, Germany; 4 Department of Neurology, Northwestern University Feinberg School of Medicine, Chicago, USA; 5 Jülich Aachen Research Alliance (JARA), JARA-Institute Molecular Neuroscience and Neuroimaging, FZ Jülich and RWTH University, Jülich, Germany; 6 Institut für Physikalische Biologie, Düsseldorf, Heinrich-Heine University, Düsseldorf, Germany; 7 Institute of Biological Information Processing (IBI-7: Structural Biochemistry), Forschungszentrum Jülich, Jülich, Germany; 8 Department of Child and Adolescent Psychiatry, Psychosomatics, and Psychotherapy, Medical Faculty, RWTH Aachen University, Aachen, Germany

**Keywords:** Glabellar Tap Reflex, Blinking, Video analysis, Camera-based, Algorithm-based, Media Pipe face mesh

## Abstract

We developed a video-based tool to quantitatively assess the Glabellar Tap Reflex (GTR) in patients with idiopathic Parkinson’s disease (iPD) as well as healthy age-matched participants. We also video-graphically assessed the effect of dopaminergic medication on the GTR in iPD patients, as well as the frequency and blinking duration of reflex and non-reflex blinks. The Glabellar Tap Reflex is a clinical sign seen in patients e.g. suffering from iPD. Reliable tools to quantify this sign are lacking. Methods: We recorded the GTR in 11 iPD patients and 12 healthy controls (HC) with a consumer-grade camera at a framerate of at least 180 images/s. In these videos, reflex and non-reflex blinks were analyzed for blink count and blinking duration in an automated fashion. Results: With our setup, the GTR can be extracted from high-framerate cameras using landmarks of the MediaPipe face algorithm. iPD patients did not habituate to the GTR; dopaminergic medication did not alter that response. iPD patients’ non-reflex blinks were higher in frequency and higher in blinking duration (width at half prominence); dopaminergic medication decreased the median frequency (Before medication—HC: p < 0.001, After medication—HC: p = 0.0026) and decreased the median blinking duration (Before medication—HC: p = 0.8594, After medication—HC: p = 0.6943)—both in the direction of HC. Conclusion: We developed a quantitative, video-based tool to assess the GTR and other blinking-specific parameters in HC and iPD patients. Further studies could compare the video data to electromyogram (EMG) data for accuracy and comparability, as well as evaluate the specificity of the GTR in patients with other neurodegenerative disorders, in whom the GTR can also be present. Significance: The video-based detection of the blinking parameters allows for unobtrusive measurement in patients, a safer and more comfortable option.

## Introduction

Idiopathic Parkinson’s Disease (iPD) is the most frequent movement disorder and severely affects patients’ quality of life [1]. Worldwide, 6.1 million individuals suffered from iPD in 2016 [2]. The prevalence increases with age, reaching a maximum in the age group between 80 and 84 years [3]. Patient numbers continue to rise due to demographic changes. iPD is a chronic progressive disorder: drugs to treat symptoms are available but treatments to stop disease progression are lacking.

iPD patients typically present pathological ways of blinking. Abnormalities are found in the blink rate, reflexive blinking, as well as the voluntary saccades [4]. Physiologically, spontaneous blinking is mainly operated by contraction of the orbicularis oculi muscle via facial nerve innervation and simultaneous relaxation of the levator palpebrae muscle [5].

In healthy individuals, blinking occurs with a frequency of 15 to 20 times per minute [6]. iPD patients typically show a lower blink rate, often associated with xerophthalmia [7–10], a dryness of the eye which iPD patients frequently suffer from. However, it has been suggested that iPD patients can be divided into two groups: one with a low-blinking-rate (mean 5.1 blinks per minute) and one with a paradoxically high-blinking-rate (mean 52.8 blinks per minute), which is assumed to be a form of off-state dystonia [11]. While blinking in iPD patients is typically hypokinetic (low blink rate), bradykinesia (in the form of increased blinking duration and decreased amplitude) does not seem to be prevalent [12].

iPD patients also often present with a pathological glabellar tap reflex (GTR). The terms blink reflex (BR), nasopalpebral reflex, orbicularis oculi reflex, or glabellar tap sign are often used synonymously [13]. The reflex, first described by Overend in 1896 [14], is a brain stem reflex for protection of the eyes that is physiologically found in neonates and can be disinhibited due to cerebral impairment as iPD disease [13, 15, 16].

Clinically the GTR presents as reflexive blinking of both eyes in reaction to a light haptic stimulus of the glabellar region, as well as acoustic, optic, or painful stimuli [17, 18]. For clinical purposes the GTR is often measured with the EMG. Electrophysiologically, the GTR can be sectioned into an initial proprioceptive and a later nociceptive component, both of which present as a blink [17].

Healthy subjects usually habituate to the GTR after the fourth tap [18]. The habituation might be caused by a decreasing inhibition of the levator palpebrae muscle [19]. Blinking up to 5 [13], 10 [15], or 15 times [5] in response to the glabella tap is considered as adaptation or habituation in the literature, which means that the GTR is not present.

iPD patients show an increased and prolonged proprioceptive and nociceptive reaction, as well as a lack of habituation of the nociceptive component [18]. This might be due to a decreased dopamine-inhibition of the striatum, as dopaminergic nigrostriatal pathways play a role in suppressing nociceptive reflexes [20]. The continued inhibition of the levator palpebrae muscle might be a result of the substantia nigra’s involvement in the pathogenesis of abnormal eye movement, such as involuntary inhibition of the levator palpebrae muscle [21].

During a task where concentration on visual input is required, the rate of spontaneous blinks decreases to allow continued visual input during voluntary saccades [6]. In iPD patients, the inhibition of blinks during voluntary saccades is decreased. Globe et al. even showed an increase in blinking frequency in iPD patients when they were asked to perform a task that required concentration [22].

Non-contact sensing modalities using cameras have emerged in the past with tremendous success in recent years [23]. Contactless measurements grant more comfortable diagnostics for patients [24]. In various fields, unobtrusive, camera-based technologies have proven successful, demonstrating their effectiveness in applications ranging from vital sign detection in neonates [24] to the identification of symptoms in neurodegenerative disorders such as progressive supranuclear palsy (PSP) and the detection of tremor in iPD patients [25, 26]. Another notable area of research is the non-contact blinking detection or tracking, for sleepiness or fatigue detection [27, 28] and analyzing the age and gender effect on blinking behavior [29]. This underscores the versatility of camera-based technologies in addressing diverse medical and physiological challenges, showcasing their potential to contribute significantly to advancements in healthcare and related research domains.

In this work, we established an algorithm that was able to detect the blink reflex in video recordings. The algorithm was able to detect the “tap” and the “blink” in the classic blink reflex maneuver, as well as provide further information on blink count and blinking duration. The video-based method can now be used to investigate the GTR in iPD patients and HC to gain further insights into the phenomenon of GTR habituation.

## Results

We developed a video-based method to detect the GTR. Therefore, we recorded the GTR of 12 HC [mean 65.7 (± 8.69) years of age] for reference and 11 iPD patients [mean 66.2 (± 8.44) years of age] on a high-framerate camera taking at least 180 images per second. This frame rate corresponds to one image every 5.6 ms. The data of eleven HC and nine patients was analyzed. This section presents the results of blink count and blinking duration of reflex and non-reflex blinks among the groups, as well as the effect of medication on these parameters in iPD patients.

### iPD patients showed no habituation to the GTR before or after medication while HC habituate after tap four

**Fig. 1:**
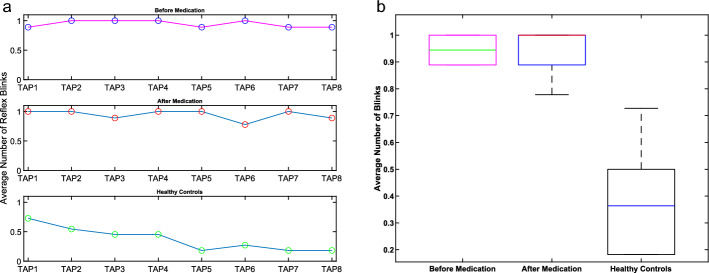
Average Number of Reflex Blinks After Taps: **a** Lineplot. We analyzed the average number of reflex blinks after taps 1–8 in iPD patients before and after medication, as well as in HC. The average number of reflex blinks did not change significantly in iPD patients before or after medication, while the average number of reflex blinks decreased from tap 1 to 8 for HC; **b** Boxplot. Here, the distributional results of the average number of reflex blinks are visualized

As shown in Table 1, for reflex blinks, there is not much change in the values for the patients between before and after medication cases. However, HC showed a decreasing average number of reflex blinks after taps. Figure 1a visualizes this result.

**Table 1:** Averaged number of blinking information

Tapping number	Average number of reflex blinks			Average number of non-reflex blinks		
	Before medication	After medication	Healthy controls	Before medication	After medication	Healthy controls
TAP01	0.888	1	0.727	0.666	2.555	0.700
TAP02	1	1	0.545	1	1.222	0.500
TAP03	1	0.888	0.454	1.333	1.777	0.400
TAP04	1	1	0.454	1.444	0.777	0.300
TAP05	0.888	1	0.181	0.888	0.666	0.300
TAP06	1	0.777	0.272	1.444	0.444	0.200
TAP07	0.888	1	0.181	1.666	0.555	0.200
TAP08	0.888	0.888	0.181	0.888	0.666	0.100
Mean (± SD)	0.944 (± 0.06)	0.944 (± 0.08)	0.374 (± 0.20)	1.166 (± 0.35)	1.083 (± 0.74)	0.338 (± 0.19)

	Reflex Blinks			Non-reflex Blinks		
Comparison	BM-AM	BM-HC	AM-HC	BM-AM	BM-HC	AM-HC
p-value	1	< 0.001	< 0.001	0.3114	< 0.001	0.0026

BM: Before medication, AM: After medication, HC: Healthy control

In Fig. 1b, patients with iPD had a similar number of reflex blinks, without difference upon L-Dopa treatment and there was no statistical difference between the two cases (p = 1). HC had a lower number of reflex blinks compared to the groups of iPD patients, which resulted in a statistical difference (Before medication-HC: p < 0.001, After medication-HC: p < 0.001).

### iPD patients had a higher count of non-reflex blinks compared to HC—after medication the median blink count decreased

**Fig. 2:**
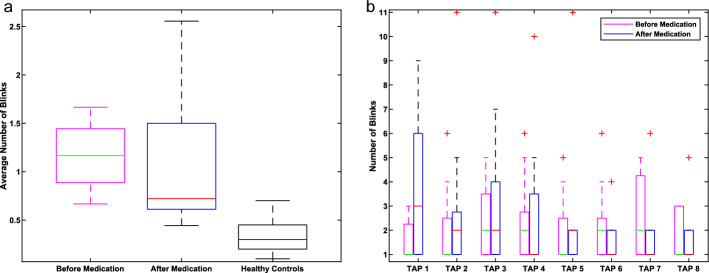
Non-Reflex Blink Count and Tap-Wise Distributions: **a** Boxplot. The figure shows the average number of non-reflex blinks after taps, comparing iPD patients (before and after medication) and HC. iPD patients showed a higher median value before and after medication compared to HC. The median value was decreased after medication, but statistically, the difference is not significant. However, the iPD patient’s results differ significantly from those of the HC; **b** Boxplot. The number of blinks of iPD patients before and after medication was calculated after each tap. Before medication, there is no in- or decrease in the number of blinks throughout the examination. After medication, iPD patients showed a higher number of blinks after the first 2–3 taps. After the third tap the median value decreased or stayed constant

For the non-reflex blinks (Table 1), Fig. 2 shows the distributional results.

iPD patients showed a higher number of non-reflex blinks and variability compared to HC (Before medication-HC: p < 0.001, After medication-HC: p = 0.0026). After medication patients had a decreased median value while variability increased. There were no differences in the iPD patients before-after L-Dopa treatment (p = 0.3114) (Fig. 2). Additional information on blink count is shown in Table 1.

### Average width of reflex blinks did not differ significantly among the groups

**Fig. 3:**
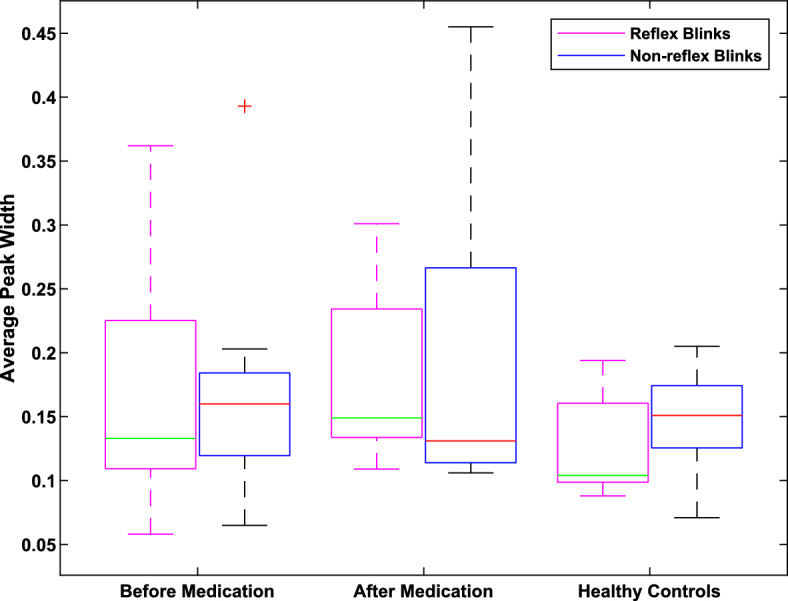
Boxplot. Patient/HC-Wise Comparison of Average Peak Width Distributions of Reflex and Non-Reflex Blinks: Reflex blinks: iPD patients had an insignificantly higher median value compared to HC; this effect is slightly enhanced by medication. Non-reflex blinks: iPD patients presented a higher median value than HC. After medication, the median value of the average width decreased

As shown in Tables 2 and 3, patients and participants showed different blinking patterns for reflex and non-reflex blinks. As for reflex blinks, iPD patients showed higher median value and variability before medication compared to HC. After medication patients experienced increased median value while variability decreased. No statistically significant difference was observed between patients and HC before and after medication (p = 0.4363, 0.1135 respectively). Figure 3 visualizes the average peak width of reflex and non-reflex blinks among the three groups.

**Table 2:** Averaged blinking width information of patients

Patient	Average width of reflex blinks (s)		Average width of non-reflex blinks (s)	
	Before medication	After medication	Before medication	After medication
1	0.115	0.173	0.137	0.141
2	0.058	0.141	0.065	0.118
3	0.362	0.301	0.393	0.455
4	0.158	0.133	0.178	0.106
5	0.120	0.149	0.203	NaN
6	0.213	0.109	0.125	0.110
7	0.092	0.134	0.103	0.138
8	0.262	0.229	0.160	0.124
9	0.133	0.250	0.176	0.392
Mean (± SD)	0.168 (± 0.1)	0.180 (± 0.07)	0.171 (± 0.09)	0.198 (± 0.14)

	Reflex Blinks	Non-reflex Blinks
Comparison	BM-AM	BM-AM
p-value	0.4501	0.8148

*NaN: there is no blinking. BM: Before medication, AM: After medication

**Table 3:** Averaged blinking width information of healthy controls

Participant	Average width of reflex blinks (s)	Average width of non-reflex blinks (s)
1	0.088	0.125
2	NaN	NaN
3	0.099	NaN
4	0.194	0.205
5	NaN	0.071
6	0.174	0.181
7	0.104	NaN
8	0.151	0.151
9	0.103	0.127
10	0.098	NaN
11	0.156	0.154
Mean ± SD	0.130 ± 0.039	0.143 ± 0.047

	Reflex Blinks		Non-reflex Blinks	
Comparison	BM-HC	AM-HC	BM-HC	AM-HC
p-value	0.4363	0.1135	0.8594	0.6943

*NaN: there is no blinking

### iPD patients showed wider (i.e. slower) non-reflex blinks than HC—medication decreased the median width of non-reflex blinks in iPD patients

As for the width of non-reflex blinks, compared to HC, iPD patients had a higher median value before medication. After medication, the median value decreased while variability increased. However, there was no statistical difference between before/after medication cases (p = 0.8148) and HC in terms of non-reflex blink widths (p = 0.8594, and 0.6943 respectively).

## Discussion

### Development of a video-based program to detect blinking parameters

We developed a videographic tool to quantitatively assess the GTR in iPD patients as well as HC. With this tool, we were able to successfully identify habituation to the GTR as well as quantify the blink count and the blinking duration. We were also able to study the effect of dopaminergic medication on iPD patients’ reflex blinking and non-reflex blinking.

### Reaction to the GTR

The results showed that the iPD patients in our cohort did not habituate to the GTR. However, the intake of dopaminergic medication did not affect the reaction to the tap in this group. Other studies suggested that dopaminergic medication can lead to a reversal of the glabellar response [31]. A study by Klawans and Goodwin showed a reversal to a normal clinical GTR response in 50% of iPD patients after being treated with L-Dopa for three months or longer [31]. The likelihood of reversing the GTR was observed to decrease with a higher Hoehn and Yahr stage as well as a longer disease duration; patients whose GTR was reversed also had a good clinical response to L-Dopa overall [31]. However, the authors studied the effect of dopaminergic medication on the GTR over a longer period of time, not the initial change after the intake of medication.

HC habituated after the fourth tap. These results are in line with the Simpson Angus Scale, in which the GTR is used as one of ten items to evaluate the severity of iPD; here up to 5 consecutive blinks after tapping are considered a normal response [32].

### Blink frequency

Before medication, the iPD patients blinked more frequently in between the taps as compared to the HC. Dopaminergic medication decreased the frequency of non-reflex blinks (closer to the HC).

As hypomimia and low blink rate are typical symptoms of iPD, we expected the iPD patients to show a lower frequency of non-reflex blinks as compared to the HC. Nevertheless, a decreased blink rate has been reported in healthy participants while performing a task that required concentration and during voluntary saccades [6, 33]. Contrary to that, Golbe et al. found an increased blink rate of iPD patients during voluntary horizontal eye movements [22]. As dopamine is associated with attention and cognitive functioning [34], reduced dopamine levels in iPD patients might affect the ability to suppress blinking during a task where concentration is required. Increased blink rate has also frequently been reported in patients with dry eye, a condition associated with autonomic dysfunction in iPD [8, 35]. In our patient cohort, a multifactorial effect on their blink rate can be assumed.

### Blink duration

We defined blinking duration as the peak width at half prominence (see Methods, Fig. 5). Concerning the reflex blinks, there was no significant difference between iPD patients and HC. When we analyzed the non-reflex blinks, iPD patients before medication blinked with a higher duration (i.e. slower) than the HC.

After medication, the blinking duration of reflex blinks increased slightly—the iPD patients’ reflex blinks slowed down after the intake of dopaminergic medication. However, the blinking duration of non-reflex blinks decreased after medication and the blinking behavior was more similar to the HC.

From a common clinical perspective, dopaminergic medication should increase the velocity (thus decrease the duration) of movements [36]. The decrease in blinking duration of the non-reflex blinks is explained by the effect of the dopaminergic medication. For the reflex blinks, we see a counterintuitive increase in blinking duration (i.e. slowing) after medication. As reflexive blinking is increased in iPD patients, the increase in blinking duration of reflex blinks after medication might be explained as a reduction of excitability of the GTR.

### Outlook

Unobtrusive measurements are regarded as safer and more comfortable [24, 37]. They do not require electrodes or any adhesives. Besides patient comfort and examination of the GTR, other scenarios of use are possible, e.g. detection of sleepiness indicated by slowing of the blink frequency in car drivers [38].

We developed a tool to quantitatively assess the GTR. This tool is contactless and easily accessible; unlike the conventional form of measurement (EMG) it does not require electrical stimulation, which makes it more comfortable for the examinee. The algorithm could be used to support early diagnosis of iPD, preferably combined with further videographic tools for common Parkinsonian symptoms such as tremor [26].

Despite our pilot sample size, we could showcase robust results of our algorithm.

For our method of measurement, there is currently no gold standard. Thus, comparison to a gold standard for accuracy is difficult. Yet, for further research, comparing the videographic assessment of the GTR to the data collected with EMG might deliver information about the accuracy of the video data and algorithm.

## Conclusions

We developed a quantitative, video-based tool to assess the GTR and other blinking-specific parameters (frequency and blinking duration) in HC as well as in iPD patients before and after medication. This tool can now be used as an easy, quick, and comfortable yet accurate method to examine blinking behavior in a clinical or scientific setting. Further studies could focus on the comparability of the video data to the EMG data of the GTR and on the applicability of this method on patient groups suffering from other neurodegenerative disorders.

## Methods

### Participants

Eleven patients with clinically unquestionable iPD [4 females (36%) and seven males (64%)] aged 52–83 years [mean 66.2 (± 8.44 Standard Deviation (SD)) years] participated in the study (see Table 4 for extended information).

**Table 4:** Extended Patient Information

Patient	Age	Sex	Years since disease onset	L-dopa equivalent Doses (mg) Daily	L-dopa equivalent doses (mg) Before the second measurement	H&Y
1	69	F	3	175	75	3
2	67	M	7	600	100	2
3	68	F	7	1125	150	2
4	69	M	20	725	125	2–3
5	52	M	17	785	75	2
6	57	F	10	1716	133	2–3
7	71	M	9	837	100	3
8	63	M	10	601	109	3
9	71	M	7	775	150	3
ex1	58	M	7	500	100	2
ex2	83	F	13	632	133	3
Mean ± SD	66,2 ± 8.44		10 ± 2.94	770.1 ± 391.3	113.6 ± 26.62	2.5 ± 0.47

Twelve healthy participants, termed HC, of both sexes [7 females (58%) and 5 males (42%)] aged 53–84 years [mean 65.7 (± 8.69) years] were enrolled. Informed consent was obtained from all participants. Extended participant information is given in Table 5.

**Table 5:** Extended healthy controls information

Participant	Age	Sex
1	53	M
2	84	M
3	77	F
4	69	F
5	61	M
6	69	M
7	63	F
8	62	F
9	67	M
10	65	F
11	64	F
ex1	54	F
Mean ± SD	65.67 ± 8.69	

Data of two patients and one participant had to be excluded from the analysis as the examiner’s finger was in the glabellar region (within the camera frame) throughout the experiment, blocking the automated tap detection.

Disease duration ranged from 3 to 20 years (mean 10 (± 2.94) years). The average Hoehn and Yahr (H&Y) stage, a scale including five stages estimating disease severity, was 2.5 (± 0.47). The patients scored with a mean of 37.9 (± 15) points in Part III of the Unified Parkinson’s Disease Rating Scale (UPDRS) (min. 22, max. 73). The UPDRS is a multiscale clinical score to describe iPD symptoms severity widely used in iPD research. While scales within the UPDRS describe several symptoms, e.g. non-motor symptoms, part III focuses on motor symptoms. One patient experienced wearing-off phenomena. All patients were in-house patients of the University Hospital Aachen enrolled in a 3-week rehabilitation program. Medication was taken according to the regular, individual scheme. The study is purely observational, and it did not interfere with the clinical treatment. The first video was recorded just before the next regular medication intake, and the second video was 30 min after. The mean L-Dopa equivalent daily dose among patients was 770.1 (± 391.3) mg. The mean dose taken before the second recording was 113.6 (± 26.62) mg.

### Video recording

We used a Lumix, GH5, Kadoma, Japan (Participant 1) and a Go-Pro HERO 7, San Mateo, CA, USA (all others). The videos were recorded in slow-motion with at least 180 frames/s). Image processing was performed with Python (Python Software Foundation) and Matlab (MathWorks, Massachusetts, USA).

We recorded the videos while examining the participants’ BR clinically (HC and iPD patients). The participants were recorded at a 45° angle. The participants were seated in a quiet, temperature-controlled room that was lit by regular artificial light, no special lighting was installed. The participants were directed to watch straight while the examiner (TJ) approached her index finger from above to the participant’s forehead (outside the visual field to eliminate visual threat as a stimulus) and tapped the region between the eyebrows 5 to 8 times (irregular rhythm, slower than 2 times per second). All participants were examined by the same examiner. The examiner was wearing a blue/blue-toned glove on her right hand that helped to distinguish the examiner’s finger from the participant’s forehead. The natural reaction to the BR was recorded in the first video.

For the iPD patients we recorded a second video 30 min after intake of their L-Dopa medication.

### Data extraction and processing

**Fig. 4:**
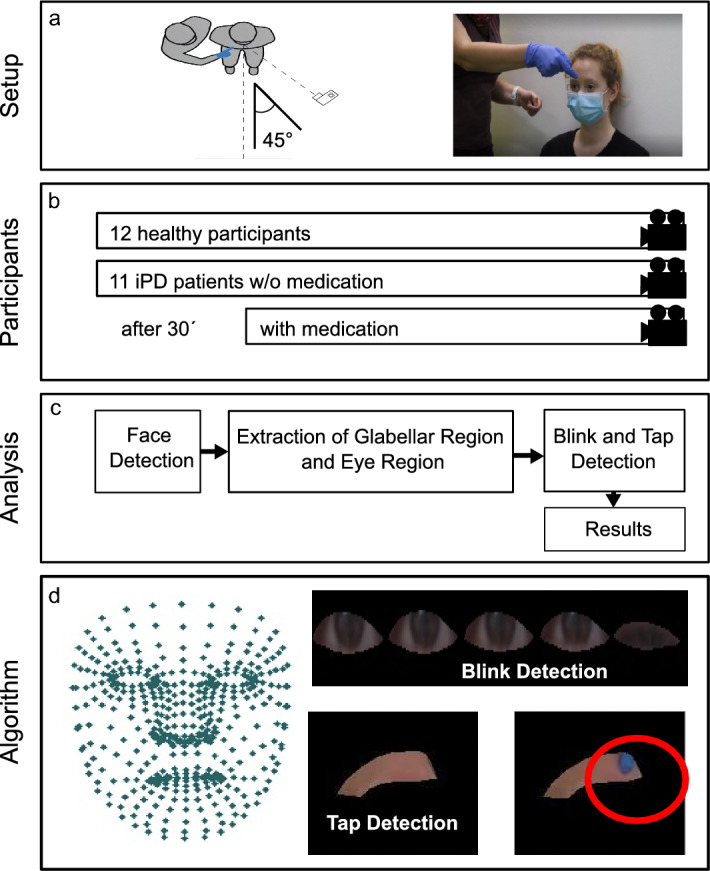
Experimental Setup: The BR was measured clinically in 12 HC and 11 iPD patients and analyzed with a high-framerate consumer camera. **a** The participants were filmed at a 45° angle, while the examiner performed the GTR. A blue/blue-toned glove was worn for better discrimination in the video analysis; **b** For the HC one video of the GTR was taken. iPD patients were examined before and 30 min after the intake of their standard L-Dopa medication. **c, d** In the next step, the MediaPipe face mesh algorithm was used for facial landmark detection. Based on the facial landmarks we defined two ROIs (Glabellar Region and Eye Region), from which information was extracted throughout the experiment, with which we were able to detect the occurrence of blinks and taps

This work aims to track and compare the eye blinking of patients (before/after medication) and HC after finger-tapping to the glabellar region, using an automated video-based approach. In this context, we used the AI-based MediaPipe face mesh [30] algorithm to track the eye blinking and the intervention in the glabellar region. The MediaPipe face mesh pipeline uses two network models that collaborate. A detector model works on the input image to compute the face locations and passes the information to the 3D face landmark model which creates the 3D mesh with 468 landmarks from those locations. A representation of 468 landmarks, each of which has three coordinates (x, y, and z), is given in Fig. 4d, left.

Based on the MediaPipe face mesh landmarks, we defined two regions of interest (ROIs). (I) The glabellar region was defined based on glabellar landmarks from the face mesh. (II) The eye ROI (left or right, depending on the recording angle) was a rectangular ROI defined by the eyelid landmarks covering the eye (pixel values outside the eye were set to zero). From the defined ROIs, the information was extracted throughout the experiment. We used two different approaches for the different ROIs. For the glabellar ROI, the blue channel of the color image was used (the examiner was wearing a blue/blue-toned nitril examination glove while applying the stimuli to make the finger more distinguishable from the skin of the participant’s forehead) and the pixels were averaged for all sequential frames to create the one-dimensional (1D) tapping signal. Without intervention by the examiner, the skin of the participant represented the average value. During the intervention, the blue glove caused a higher average value, so that we were able to detect the timepoint when the tap to the glabella occurred. Sample images of tracked glabellar ROI with and without intervention are given in Fig. 4d.

The landmarks from the face mesh algorithm have the sensitivity to track the eyelids. We used the eye ROI based on the eyelid contours and tracked this ROI for all frames. During the tracking, the number of non-zero pixels was counted for all frames and then inverted to create a 1D signal with positive amplitude. When there was no eye-blink, the number of non-zero pixels was relatively high. During the lid closure, the eye ROI shrank down and led to a lower number of non-zero pixels. A sample image of tracked eye ROI is given in Fig. 4d.

**Fig. 5:**
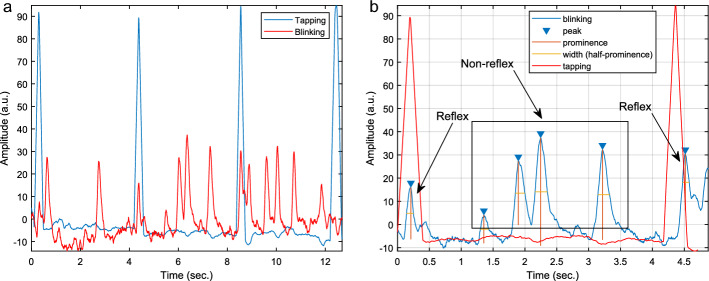
Example Tapping and Blinking Signal: **a** Line plot. Tapping and blinking signals are visualized throughout the examination of the GTR. Blue peaks show the tapping of the glabella region on the participant’s forehead while red peaks visualize when blinks were detected; **b** Line plot. To differentiate blinks as a response to the tapping of the glabella from other blinks, blinks were divided into two groups: reflex blinks, which occurred in the time frame from 50 ms before until 200 ms after the tapping signal, and non-reflex blinks in between two consecutive taps, but outside of the aforementioned time range. The blinking duration was defined as the width of each blink, which was calculated at half prominence. a.u. = arbitrary unit

Next, the signal was set to a common baseline by applying baseline removal of all 1D signals. Following the baseline correction, data were low-pass filtered at 0.75 Hz cut-off frequency. The frequency was determined empirically, to reduce flickering in illumination. An example of extracted 1D tapping and blinking signals are given in Fig. 5a.

We defined two different blink categories. (I) The reflex blinks which occurred 50 ms before to 200 ms after the tapping signal and (II) the non-reflex blinks which occurred between two consecutive taps.

For further blink analysis we defined the blinking duration as width at half of the maximum prominence (half-prominence) and the number of reflex/non-reflex blinks was counted. Figure 5b shows the example of reflex and non-reflex signals with the calculation of width at half-prominence.

### Statistical analysis

Manual curation of each video was compiled in a database (Microsoft Excel version 16.42 [Microsoft, Redmond, Washington, USA]). All computations were performed using MATLAB R2019b, The MathWorks, Natick, MA, USA. Descriptive statistics were applied. Normal distribution of data was tested by the Shapiro–Wilk tests. As it was impossible to transform the non-normal data using neither x^2^, 1/x, root nor ln, a non-parametrical test (Wilcoxon rank-sum test, as calculated with MATLAB R2022b) was applied to determine differences between groups. Significance was defined as p < 0.05.

## Data Availability

Data sharing is not available. Videos cannot be shared due to privacy reasons. Extracted primary data are shown in supplementary tables.

## Abbreviations

GTR: Glabellar Tap Teflex
iPD: Idiopathic Parkinson’s Disease
HC: Healthy controls
EMG: Electromyogram
BR: Blink Reflex
PSP: Progressive Supranuclear Palsy
H&Y: Hoehn and Yahr
UPDRS: Unified Parkinson’s Disease Rating Scale
ROIs: Regions of Interest
1D: One-dimensional
SD: Standard Deviation
